# The Well-being and Instructional Experiences of K-12 Music Educators: Starting a New School Year During a Pandemic

**DOI:** 10.3389/fpsyg.2021.701189

**Published:** 2021-07-21

**Authors:** Kelly A. Parkes, Joshua A. Russell, William I. Bauer, Peter Miksza

**Affiliations:** ^1^Teachers College, Columbia University, New York, NY, United States; ^2^The Hartt School, The University of Hartford, West Hartford, CT, United States; ^3^School of Music, University of Florida, Gainesville, FL, United States; ^4^Jacobs School of Music, Indiana University, Bloomington, IN, United States

**Keywords:** music teachers, music education, well- and ill-being, COVID-19, pandemic, depression, PERMA, DASS-21

## Abstract

In adapting to remote emergency teaching modes during pandemic-imposed conditions, teachers’ instruction has changed dramatically. Early research indicates that the well-being of music teachers has suffered during the COVID-19 pandemic and that high levels of depression are widespread. The purpose of this survey study was to assess the continued psychological well-being of music teachers working amid a global pandemic based upon previous research we conducted during the Spring 2020 semester when most teachers in the United States were forced into emergency remote teaching. A secondary purpose was to explore the ways that pandemic conditions have affected music teachers’ sense of safety at work and their current teaching situations. Our questionnaire consisted of sections pertaining to (1) demographic and institutional information, (2) well-being and depression, (3) instructional format and preparedness, (4) teaching efficacy compared to the start of the pandemic, and (5) potential positive outcomes of the pandemic-imposed adjustments. In total, 1,325 music teachers responded to our survey. Overall, the participants reported poorer well-being than both published norms and the sample of participants in our previous study. In addition, 17% reported mild depression, 25% reported moderate depression, and 24% reported severe extremely severe levels of depression. Summaries of the participants instructional experiences and their implications for music education are discussed within.

## Introduction

Educators across the United States have been teaching remotely in response to the COVID-19 pandemic since early 2020 (MCH Strategic Data, 2020), a period so dire that in the United States, life expectancy has reportedly dropped by a full year (Wamsley, 2021). Teachers^1^ are anxious, considering leaving the field, and yet remain committed to their students (Educator Voice, 2020; Goldstein and Shapiro, 2020). Music teachers have adapted to remote teaching and learning (Hash, 2021); however, early research indicates that the well-being of music teachers has suffered during the COVID-19 pandemic and high levels of depression have been reported (Miksza et al., 2021; Parkes et al., 2021). Teaching, in general, can be considered a stressful profession (MetLife, 2013; Gray et al., 2017), and for music teachers, there are specific stressors, such as finding a balance between personal and professional life demands, the inclusion of job tasks not related to music, a lack of resources, and the experience of role overload (Scheib, 2003). Isolation is also a recognized factor in the levels of stress music teachers experience (Hedden, 2005), and during pandemic-imposed conditions, the number of stressors has increased as teachers report a loss of income, difficulty maintaining boundaries between professional and personal life, social isolation, exhaustion, the use of new technologies, grief of losing a previous way of life, and apprehension about the future (Cornett, 2020). In adapting to remote emergency teaching during pandemic-imposed conditions, teachers’ instruction has changed dramatically, and concomitantly, their well-being has suffered.

### Well-being

Seligman’s (2011) well-being theory has been used in music-related research, with professional musicians (Ascenso et al., 2018) and collegiate musicians (Roseth, 2019). Seligman’s (2011) model of well-being comprises five elements: Positive Emotion, Engagement, Relationships, Meaning, and Accomplishment (i.e., the PERMA model). The criteria for elements of the PERMA model require that they (1) contribute to well-being, (2) are something that individuals generally pursue for its own sake, and (3) can be defined and measured relatively independently. Positive Emotion indicates general feelings of positive affect and feeling well. Engagement indicates feelings of absorption in and/or connections with tasks individuals pursue, and the Relationships element indicates social connections to other individuals and beliefs that one is loved and cared for. Meaning indicates a sense that one’s life matters and that they are part of something larger than themselves. Finally, Accomplishment indicates the perception that one is achieving goals, with an emphasis on intrinsically defined goals. The well-being of music teachers in the United States has been examined with this model (Miksza et al., 2021; Parkes et al., 2021), and the findings indicated significantly lower levels of overall well-being, in addition to significantly higher levels of depression than published norms.

Cheng and Lam (2021) studied the well-being of music teachers in Hong Kong. Some of the teachers (*n* = 20) in their sample were interviewed, whereas others (*n* = 120) completed a survey which included a Fear of Coronavirus-19 Scale, a Generalized Anxiety Disorder Scale, and a Chinese Teacher Stress Questionnaire (short form). Survey data were analyzed to identify psychological impacts and behaviors linked to the pandemic. Cheng and Lam (2021) found that Hong Kong teachers were not coping well with the pandemic-imposed changes. The findings showed that music teachers were experiencing discomfort, fear, nervousness, and anxiety in response to the pandemic. Within the survey data, teachers reported high levels of stress around the effectiveness of online music teaching, parental expectations, and students’ adaptability to online learning. In addition, challenges of technological integration and maintaining professionalism during this period of transformative teaching also seemed to cause anxiety.

### Pandemic Instruction

Hash (2021, p. 384) suggested that the first 6 months of remote learning in Spring 2020 was “emergency teaching” rather than online distance learning (ODL). Hodges et al. (2020) noted that emergency remote teaching is different to carefully designed ODL. In his study, Hash (2021) reported that band directors (*n* = 462) faced several challenges, seen more often in schools in rural locations and/or with higher poverty levels. Hash noted that there were some affordances, with teachers using a greater variety of technology, being able to focus more on individual student achievement, greater variety of musical content (such as music theory, history, and culture) along with an opportunity for more student creativity (such as arranging and composition). Hash also found that almost all the teachers in his study (*n* = 459, 99.4%) indicated that maintaining students’ well-being was a high priority. Although it was not reported in the study, it may be reasonable to question how maintaining students’ well-being may have added to the stress teachers absorb while teaching in pandemic-imposed conditions.

In addition to Hash’s (2021) work in the United States, there are a few researchers who have examined music teachers’ instructional experiences in other countries. Daubney and Fautley (2020, 2021) suggested that music education in England during the COVID-19 pandemic could be likened to “u-turns in the fog” (Daubney and Fautley, 2021, p. 3) and that since the teaching professionals were holding communities together, educating young people, and feeding families in the most challenging of times, “they might also bring renewed respect for the profession” (Daubney and Fautley, 2021, p. 10). Their description of music teachers is positive but perhaps does not consider the collateral damage teachers may have experienced as they navigated remote teaching. In Australia, de Bruin (2021) explored the pedagogical practices and behaviors of 15 instrumental music teachers over 8 months of isolated remote learning. He found that the development of relatedness between teacher and student was critical. Relatedness was important not only for helping students connect with musical experiences, but also to the teacher. de Bruin suggested that strong forms of connection, social expression, and relatedness were important in students’ engagement, as well as student emotional engagement and well-being. He noted that emotional support was provided for students by teachers and that it was the teacher’s ability to create new learning relations, levels of understanding, and a sense of reciprocity that allowed students to feel comfortable in their remote lessons. de Bruin’s research was focused on the pedagogical practices of teachers, so it is not clear what impact these practices may have had on the teachers’ own well-being.

Daugvilaite (2021) reported the findings from a holistic case study that 10 young students learning online in London became more independent over the course of the 4 months. However, students and their parents also reported that the physical absence of the teacher had negatively impacted their learning, citing the lack of non-verbal communication, gestures, scaffolding, and a tactile approach as influential factors. In addition, the absence of the teacher also negatively impacted the students’ levels of engagement and motivation. In Spain, Calderón-Garrido and Gustems-Carnicer (2021) reported that of the 355 teachers they surveyed, most preferred to continue teaching during the emergency. They also reported that teachers did not have access to methodological and material resources. Many of the teachers noted a lack of specific instructions from their superiors for emergency remote teaching, and the researchers reported differences between public, private, and semi-private school teachers’ perspectives. The teachers in this study reported that teaching asynchronously online had allowed them more contact with students.

In a joint publication by authors in the network of Music Teacher Associations in Europe (Pabst-Kruger and Ziegenmeyer, 2021), prevalent themes emerge: first, the reactions of teachers to online learning; second, the challenges faced by teachers; and third, possible benefits afforded by online instruction in music education. Gibson (2021) conducted a case study of five Ethno-artistic mentors—three Indians, one American, and one from Bosnia–Herzegovina—to determine their perceptions of the shift to online music making. She found in the mentors’ perceptions both an acceptance of the affordances of online learning as well as a recognition of the irreplaceable features of in-person learning environments as part of their engagement in a series of 40 live-streamed sessions of situated learning and music making.

### Efficacy and Professional Development

Black et al. (2020, p.119) suggested that in general, “teachers were unprepared and untrained to handle the complexities inherent to educating as well as the demands of the technology needed to support these efforts. Although teachers deserve high praise for their rapid response, the educational outcomes were unsatisfying.” We suggest that this may be due to teachers’ diminished perceptions of their own efficacy. A teachers’ sense of teaching efficacy has been defined as a teacher’s “judgment of his or her capabilities to bring about desired outcomes of student engagement and learning, even among those students who may be difficult or unmotivated” (Tschannen-Moran and Woolfolk Hoy, 2001, p. 783). The teaching efficacy construct has been linked with important outcomes for teachers, including well-being (Smylie, 1988; Tschannen-Moran and Woolfolk Hoy, 2001; Egyed and Short, 2006). Teachers’ experience of stress has been reported as an important contributor to their sense of teaching efficacy (Klassen and Chiu, 2011) as well as how they feel about school climate (Collie et al., 2012). Several researchers, such as Shann (1998), Wilson (2002), and Tschannen-Moran and Woolfolk Hoy (2007), have indicated that teacher efficacy, stress, and job satisfaction are related. These three variables affect not only teachers but also students directly (Collie et al., 2012).

Teachers’ connectedness to students and colleagues has been recognized as an additionally salient element, along with efficacy. Klassen et al. (2012) reported links between efficacy and teaching engagement, emotional exhaustion, and psychological distress of teachers. Feelings of burnout can contribute to lower levels of well-being, and teacher burnout has consequences for students (Madigan and Kim, 2021). Teachers’ well-being may also be mediated by levels of professional development or staffing support (Smylie, 1988). Flook et al. (2013) explored the ways in which mindfulness mediated stress, burnout, and teaching efficacy. They reported that mindfulness courses, received by teachers as professional development, led to reductions in the psychological symptoms of burnout and enhanced self-compassion.

Specifically in music education, the professional development of teachers has been difficult due to the nature of balance between teaching work activities with educational opportunities (Biasutti et al., 2019). Biasutti et al. (2019) reported that online professional development holds the most potential for music teachers yet with the move to online teaching during the pandemic, screen fatigue may prevent music teachers from engaging in development provided in online sessions. Professional development in music educators has been examined in early career teachers (Conway, 2007), experienced teachers (Bauer, 2007), and teachers in the later part of their careers (Russell, 2012; Eros, 2013), and it is clear that teachers’ needs for professional development change over the course of their careers (Conway, 2008). It therefore stands to reason that teachers might need pandemic-specific professional development while teaching music during a pandemic.

Norris (2020) suggested that there was much that teachers could do to collaborate, innovate, and find a way forward with pandemic pedagogy, but perhaps, this suggestion neglects the notion that teachers may not have had professional or psychological supports in place to continue pandemic pedagogies or engage in professional development to improve their pandemic pedagogies. In their report suggesting ways in which teachers can improve, Fullan et al. (2020, p.13) argued broadly that all teachers need to establish for their students a learning environment that focuses on well-being and belonging and that teachers should “ease the social pathway for students” with the following recommendations:

- Facilitating connection and conversation
- Re-creating norms that will allow students to feel psychologically safe in an optimistic and efficacious learning environment
- Inviting each student’s perspective by asking open questions so that each student feels connected to the learning community
- Providing trauma-informed learning for staff, parents, and students, enabling everyone in the school community to recognize and respond mindfully during this crisis
- Appoint a caring adult to build a relationship with those students … know[n] to be vulnerable (p. 13)

There are no recommendations given for teachers themselves who perhaps have not experienced connection and conversation, who are not feeling psychologically or physically safe, who are not connected to their professional development learning community, who have not had their own trauma recognized, or who have not been assigned caring administrators/mentors with whom they have built relationships.

### Research Problem, Purpose, and Questions

As the current global health crisis continues to perpetuate ongoing changes and stressors in music educators’ lives, it is imperative to continue to examine the well-being of music educators. Although the impact of the stressors of different learning formats and working conditions may have evolved as music educators have gained experience and knowledge in digital/remote teaching, an ongoing examination of the well-being of music educators is necessary to better understand how to serve in-service music educators and subsequently their students. As such, the purpose of this descriptive study was to assess the continued psychological well-being of music teachers working amid a global pandemic based upon previous research conducted during the Spring 2020 semester when most teachers in the United States were forced into emergency remote teaching. A secondary purpose was to explore the ways that pandemic conditions have affected music teachers’ sense of safety at work and their current teaching situations. To achieve these purposes, we asked the following research questions:

1. What were music teachers’ perceptions of their well-being while carrying out their teaching duties amid continued pandemic-imposed disruptions in the Fall of 2020?
  - How did music teachers’ perception of well-being vary when considering selected individual characteristics, such as student grade-level taught, teaching emphasis, years taught, or majority student ethnicity?
  - How did music teachers’ perception of well-being vary when considering selected characteristics of their schools, such as school setting, school Title I status, or school size?
2. What modes of instructional delivery were music teachers asked to carry out, how prepared did they feel to teach in the various modes, and what is their perception of the impact of the various modes upon student learning?
  - Did music teachers’ perception of acclimation vary when considering whether they had professional development?
  - Were music teachers’ perceptions of risks to their health related to their well-being?
3. What were music teachers’ perceptions of the resources available to them and the expectations made of them as compared to other teachers at their schools?
4. How have music teachers’ perceptions of their teaching efficacy changed since the Spring of 2020 and do they perceive any positive outcomes emerging from the changes in their instructional practices after gaining more experience?
  - Were music teachers’ perceptions of changes to their teaching efficacy related to their well-being?

## Materials and Methods

### Sampling and Participants

Following the receipt of IRB approval of our study, we solicited the assistance of the National Association for Music Education (NAfME) to help us survey their membership of elementary and secondary music educators in the United States. As such, we employed the membership of the NAfME as the sampling frame for this study. The NAfME sent email invitations at the end of November 2020 to their members (NAfME, 2021) requesting their participation in this research on behalf of the researchers, targeting individuals who indicated in their membership information that they taught elementary or secondary music of any kind (e.g., general music, concert band, marching band, orchestra, contemporary instruments, small ensemble, piano, guitar, chorus, vocal ensemble, and music theory).

### Procedures

Of the 25,817 email invitations sent, 18,473 were not opened and 882 bounced back, leaving 6,462 that were opened and 822 that were clicked through (i.e., they accessed a link to our online questionnaire). In a follow-up invitation in mid-December 2020, NAfME staff members sent 25,782 email invitations to the same potential participants as in the first invitation (number difference based upon changes in membership). In this mailing, 17,184 music educators did not open the invitation and 878 bounced back, leaving 7,720 that were opened and 829 that were clicked through. Ultimately, 1,325 volunteers responded to the questionnaire. As such, the response rate was approximately 5% when considering all possible members contacted, whereas the percentage of those who completed the survey compared to those who received and opened the email was 17–21%. Even with the smaller response rate based on all potential participants from the NAfME mailing, we achieved a 3% margin of error at 95% confidence (Scheaffer et al., 1995; Cochran, 2007).

### Typical PK-12 Music Teacher Profile

The typical participant in this study was White/Caucasian (91%, followed by Hispanic/Latino 2.1%, Black/African-American 1.8%, and Asian/Asian-American 1.5%), had taught for 16 years (*SD* = 10.48), and self-identified as female (67.1%, followed by 32.7% male, and only one respondent identifying as transgender and one as non-binary). The majority of participants had earned a master’s degree (57.9%), whereas 38.6% held a bachelor’s degree and only 3.3% had earned a doctoral degree. The vast majority of the participants had earned certification to teach in their state *via* a traditional 4- or 5-year teacher preparation program (91.1%). A traditional teacher preparation program in the United States begins in the undergraduate level of university where students develop their musicianship and pedagogical teaching skills, graduating with a teaching certificate/license either at the end of a baccalaureate degree (4 years), or undergraduate and graduate degrees (5 years). Few participants (7%) earned their certification through an alternate route, while very few reported not being certified to teach in their state (1.8%). This participant profile is highly aligned with a recent study using the same population regarding music teacher well-being (Miksza et al., 2021; Parkes et al., 2021).

### Participants’ Teaching Contexts

The vast majority of participants reported working in a school in which the majority of students were white/Caucasian (76.7%) followed by Hispanic or Latino(x) (10.0%), and Black or African-American (7.4%). The proportions of participants teaching at the elementary (28.5%), middle school/junior high school (31.1%), and high school (40.1%) levels were similar. Most of the participants reported being either a band director (35.3%) or a general music educator (25.6%) followed by choir director (21.2%). Relatively, few orchestra directors participated (10.4%), while the remaining teachers indicated their area of teaching as “other” (6.2%).

The majority of participants taught in suburban settings (52.9%) followed by rural settings (31.0%) and urban settings (16.0%). Nearly half of the schools in which participants taught were Title 1 schools (45.8%). Title 1 schools are schools in which children from low-income families make up at least 40% of enrollment (United States Department of Education, 2021). Similarly, nearly half of the schools in which the participants taught were middle-sized schools (between 501 and 1,200 students, 45.1%) followed by small schools (fewer than 500 students, 34.2%) and then large schools (1,201 students or more, 20.6%). Again, these teaching contexts are highly aligned with the preceding study of the PK-12 NAfME membership (Miksza et al., 2021; Parkes et al., 2021).

### Questionnaire Design

For this study, we employed elements of a researcher-created questionnaire that has been used in previous research (Miksza et al., 2021; Parkes et al., 2021) as well as established scales of well-being. We used these instruments in order to be able to more accurately explore the evolution of music educators’ well-being from the time of Spring 2020 to the current academic year. We updated this version of the questionnaire to consist of two of the original sections, i.e., (1) demographic information and institutional information and (2) music teacher well-being. We then designed two new sections of the questionnaire to address (3) instructional format and preparation and (4) instructional experiences. The second section of the questionnaire focused on teacher well-being; we employed scales from two different established measures—the PERMA Profiler (Butler and Kern, 2016), which was built upon Seligman’s (2011) theory of the elements of human flourishing (i.e., positive emotion, engagement, relationships, meaning, and accomplishment), and the 7-item depression scale from the DASS-21 instrument (Depression, Anxiety, and Stress Scale; Lovibond and Lovibond, 1995). This sub-scale was designed to assess individuals’ emotional state of depression with items addressing the following issues: dysphoria, hopelessness, devaluation of life, self-deprecation, lack of interest/involvement, anhedonia, and inertia.

In order to explore music educators’ instructional format and preparation, we constructed items to asking about what format of instruction was being used (e.g., fully face-to-face, hybrid, and online), at what point in the year the school decided upon the instructional format for music teachers and whether or not the format had changed this year and how much time teachers were given to transition to any changes in teaching format. We also asked participants about the degree to which they believed different stakeholders influenced decisions about the format of instruction in their school (e.g., teacher preference, student preference, national directives, state directives, childcare needs, economic concerns, and political opinions).

We asked music teachers to compare aspects of their teaching experience to other non-music teachers in their buildings, such as class size, teaching space, parental expectations, administrative expectations, and technological support. Additionally, we asked participants to compare their current teaching experiences to those of Spring 2020 in which most teachers were moved to emergency remote teaching due to the global pandemic. Finally, this section of the questionnaire included items designed to elicit information regarding participants’ opinion of the efficacy of the professional development they received to prepare to teach in various formats as well as any positive outcomes due to the pandemic.

### Music Teacher Well-being

#### PERMA Profiler

We included the Positive Emotion and Health sub-scales from the PERMA profiler (Butler and Kern, 2016) in the questionnaire as measures of participants’ well-being. Each sub-scale includes three items that are rated by respondents using 11-point, Likert-type scales. The response options for the items vary according to the wording of each item; for example, some items are responded to using a scale of 0—Not at All to 10—Completely, whereas others are paired with a scale of 0—Never to 10—Always, and others still are paired with a scale of 0—Terrible to 10—Excellent. Scores for each sub-scale are calculated by averaging the item responses to each 3-item collection, with low scores indicating low levels of the presence of each construct and vice versa. For the current study, participants responded to each item after reading the following prompt:

Considering the impact of your recent experiences teaching during pandemic-imposed disruptions, please answer the following questions about your current sense of well-being using the scales provided. There are no right or wrong answers. Do not spend too much time on any statement.

The internal consistency of these scales was assessed with Cronbach’s alpha; both scales had alpha values of 0.91.

#### Depression Scale

The 7-item depression scale from the short-form of the Depression, Anxiety, and Stress Scale (DASS-21; Lovibond and Lovibond, 1995) was also included in the questionnaire. The items are designed to assess participants’ emotional state of depression with items addressing the following issues: dysphoria, hopelessness, devaluation of life, self-deprecation, lack of interest/involvement, anhedonia, and inertia. Each item is paired with a Likert-type scale with the following response options: 0—Did Not Apply to Me At All, 1—Applied to Me to Some Degree, or Some of the Time, 2—Applied to Me to a Considerable Degree or a Good Part of the Time, and 4—Applied to Me Very Much or Most of the Time. Participants’ depression scores were created by summing the responses to all the items, with low values indicating very little sense of depression and high values indicating more severe depression. The total score was then multiplied by two so that score yielded from the short form of the measure can be interpreted in manner comparable to the long form of the measure. The Cronbach’s alpha estimate of internal consistency for the scale was 0.90. Participants responded to each item after reading the following prompt:

Please read each statement and adjust the slider to the number 0, 1, 2 or 3 which indicates how much the statement applied to you while teaching during the pandemic-imposed disruptions. There are no right or wrong answers. Do not spend too much time on any statement.

## Results

### What Were Music Teachers’ Perceptions of Their Well-being While Carrying Out Their Teaching Duties Amid Continued Pandemic-Imposed Disruptions in the Fall of 2020?

On average, participants reported experiencing relatively moderate degrees of Positive Emotion (*M =* 5.27, *SD* = 2.02) and Health (*M =* 6.17, *SD =* 2.15; Table 1). However, when compared to published norms for these scales, the participants in our study reported significantly less Positive Emotion, *t*(1254) = 24.89, *p <* 0.001, *d =* 0.70, and significantly poorer Health, *t*(1254) = 12.73, *p <* 0.001, *d =* 0.35. Moreover, the participants in the current study also reported significantly less Positive Emotion, *t*(1254) = 8.22, *p <* 0.001, *d =* 0.23, and significantly poorer Health, *t*(1254) = 5.00, *p <* 0.001, *d =* 0.14, than a sample drawn from the same population approximately 5 months earlier (i.e., at the end of the previous school year).

**Table 1:** Descriptive statistics for well-being and Depression scales.

	*M*	*SD*	Skew	Kurtosis	25th %	50th %	75th %
Positive Emotion	5.27^a^	2.02	−0.09	−0.74	3.66	5.33	6.83
Health	6.17^a^	2.15	−0.27	−0.68	4.66	6.33	8.00
Depression	15.72^b^	9.56	0.53	−0.32	8.00	14.00	22.00

a Range of possible scores is 0–10.

b Range of possible scores is 0–48.

According to the criteria set in the DASS-21 manual, it was evident that 23% of the sample had scores indicative of no depression (i.e., < 9), 17% reported mild depression (i.e., between 10 and 13), 25% reported moderate depression (i.e., between 14 and 20), 12% had scores indicative of severe depression (between 21 and 27), and 12% had scores indicative of extremely severe depression (i.e., >28). The difference between the mean Depression score for the participants in the current study and that of the previous sample was not significant, *t*(1206) = 2.09, *p =* 0.03, *d =* 0.06.

### Well-being and Individual Teacher Characteristics

We also examined whether our participants’ perceptions of well-being varied as a function of selected individual characteristics. A multivariate analysis of variance was conducted with the Positive Emotion, Health, and Depression scores serving as the combined dependent variable and (1) student grade-level participants taught, (2) their teaching emphasis area, (3) self-reported gender (male and female were the categories for this variable since only two participants indicated otherwise), and (4) the number of years they have been teaching serving as the independent variables. We also included the interaction of student grade-level participants taught and self-reported gender in our model since a similar effect was found in our previous research (Miksza et al., 2021; Parkes et al., 2021). Two multivariate outliers were identified and removed for these analyses; however, all other assumptions were met. In addition, the teaching emphasis variable was recoded such that the following categories were considered: general music, band, choir, orchestra, and other. Significant multivariate effects were found for teaching emphasis and years taught (Table 2). However, partial eta-squared values indicated that the effect of teaching emphasis and years were both small in magnitude, $\eta_{p}^{2}=0.010$ and $\eta_{p}^{2}=0.057,$ respectively.

**Table 2:** Multivariate regression analysis of Positive Emotion, Health, and Depression as a function of teacher characteristic variables.

Source	Pillai’s Trace	df	df error	*F*	*p*	$\eta_{p}^{2}$
Student grade level^a^	0.001	6	2,332	0.33	0.32	0.002
Teaching emphasis^a^	0.031	12	3,501	3.13	<0.001	0.010
Years taught^b^	0.057	3	1,165	25.59	<0.001	0.057
Gender^a^	0.001	3	1,165	0.14	0.93	0.002
Gender X student grade level	0.002	6	2,332	0.57	0.75	0.001

a Entered as nominal-level predictor.

b Entered as interval-level predictor.

Univariate analyses of variance were conducted for each of the well-being outcome variables and with the participants’ teaching emphasis area and number of years they have been teaching serving as independent variables. Games–Howell tests with *p-*values adjusted for multiple comparisons were used to conduct pairwise comparisons between the various teaching emphasis categories. For the Positive Emotion scale, years of teaching was positively related to reports of Positive Emotion and accounted for 4% of the variance. Teaching emphasis only accounted for 1% of the variance across Positive Emotion scores with general music teachers (*M* = 5.44, *SD* = 2.02) and orchestra teachers (*M* = 5.76, *SD* = 2.01) reported significantly stronger feelings of Positive Emotion than participants categorized as “other” (*M* = 4.78, *SD* = 2.12) for their teaching emphasis.

Regarding the Health scale, teaching explained 2% of the variance across scores. Orchestra teachers (*M* = 6.87, *SD* = 2.04) reported significantly better sense of Health than general music teachers (*M* = 6.09, *SD* = 2.08), band teachers (*M* = 6.09, *SD* = 2.10), and those categorized as other (*M* = 5.40, *SD* = 2.38). In addition, choir teachers reported significantly worse sense of Health than teachers categorized as other. Years of teaching were also positively related to participants’ reports of Health, but this variable only explained 1% of the variance in Health scores.

Years of teaching was negatively related to Depression scores and accounted for 5% of the variance. Depression scores also varied significantly as a function of teaching emphasis with band teachers (*M* = 8.09, *SD* = 4.77) reporting more depression than orchestra teachers (*M* = 6.78, *SD* = 4.46); however, this effect only accounted for 0.9% of the variation in Depression scores.

### Well-being and Teacher’s School Characteristics

We conducted analyses to determine whether music teachers’ perceptions of well-being varied as a function of selected characteristics of their schools as well. Similar to our previous analyses, a multivariate regression analysis was conducted with the Positive Emotion, Health, and Depression scores serving as the combined dependent variable and (1) school urbanicity, (2) whether their school qualified for Title 1 status, and (3) school size serving as the independent variables. The two multivariate outliers mentioned earlier were removed for these analyses as well and, again, all other assumptions were met. No significant multivariate effects were found for any of the independent variables.

### What Modes of Instructional Delivery Were Music Teachers Asked to Carry Out, How Prepared Did They Feel to Teach in the Various Modes, and What Is Their Perception of the Impact of the Various Modes Upon Student Learning?

Most of our participants began their school year in a fully online instructional mode (*n* = 454, 37.9%), with the remaining teachers split fairly evenly across the other options we provided in our questionnaire; fully face-to-face (*n* = 200, 16.7%), hybrid (part-time online and part-time face-to-face; *n* = 182, 15.2%), mixed (some students online and some face-to-face; *n* = 176, 14.7%), and simultaneous students online and face-to-face (*n* = 150, 12.5%). Only 3% of teachers (*n* = 36) chose the option of other for this item. Overall, the participants were not given much forewarning about the mode of instruction they would begin their year in with only 14% (*n* = 169) being informed 5 weeks or more before the start of the year. Participants reported whether they felt they had adequate time to prepare for the start of the school year by rating a statement using a Likert-type scale ranging from 1 *strongly disagree* to 10 *strongly agree*, which results in a median rating of 4 (*IQR* = 4).

We also asked participants to report whether they had engaged in any professional development activities over the summer months to help prepare them for the start of the school year and, if so, to rate how helpful they felt it was. Nearly three out of every four participants had taken part in some kind of professional development experience (*n* = 842, 73.3%). On a scale ranging from 1 *not at all helpful* to 10 *extremely helpful*, the participants tended to report that the professional development was somewhat helpful (*Mdn =* 6, IQR = 4). Those who had participated in professional development also had an opportunity to describe what the experience entailed. Professional development experiences centered on technology generally (e.g., Google classroom, Zoom, and Canvas; *n =* 503, 52.0%) or music technology (e.g., QuaverMusic, Soundtrap, and Sight Reading Factory; *n =* 288, 29.8%), specifically, were the most frequently reported. Other topics mentioned included methods for supporting students and themselves (e.g., mindfulness, wellness, working with special needs students, and social emotional learning; *n =* 82, 8.5%), safety procedures (e.g., cleaning, social distancing guides, science, or aerosols; *n =* 45, 4.6%), and education regarding social justice-related themes (e.g., equity and anti-racism education; *n =* 35, 3.6%). For the relatively few who reported the length of their experiences (*n =* 194, 23.0%), it appeared that the opportunities tended to be relatively short (i.e., approximately 1 week or less in duration; *n =* 164, 84.5%).

Nearly 60% of the participants (*n =* 716, 59.8%) reported that their mode of instruction was changed following the start of the school year. Among these participants, the most common mode of instruction following the change was teaching fully online (*n =* 228, 33.1%), followed in descending order by simultaneous students online and face-to-face (*n* = 116, 16.9%), mixed (some students online and some face-to-face; *n =* 108, 15.7%), hybrid (part-time online and part-time face-to-face; *n* = 102, 14.8%), fully face-to-face (*n =* 73, 10.6%), and other (*n* = 61, 8.9%). These participants were given very little time to prepare for this change; only about 27% (*n* = 189) were given more than 4 weeks to prepare, and about 35% (*n =* 238) were given only 1 week to prepare. Participants reported whether they felt they had adequate time to prepare for the change in mode of instruction by rating a statement using a scale ranging from 1 *strongly disagree* to 10 *strongly agree*, which resulted in a median rating of 4 (*IQR* = 4).

All participants also indicated whether they believed students in either face-to-face or online instruction were receiving a better educational experience. Most reported that students in face-to-face instruction yielded a better experience (*n =* 662, 56.8%), whereas hardly any participants believed online instruction to be the better experience (*n* = 25, 2.2%). In addition, nearly 10% of participants (*n =* 115) thought that both modes of instruction were comparable and about one third of the participants (*n =* 362) indicated that they could not make a decision since they only had experience with one of the modalities or the other.

**Figure 1:**
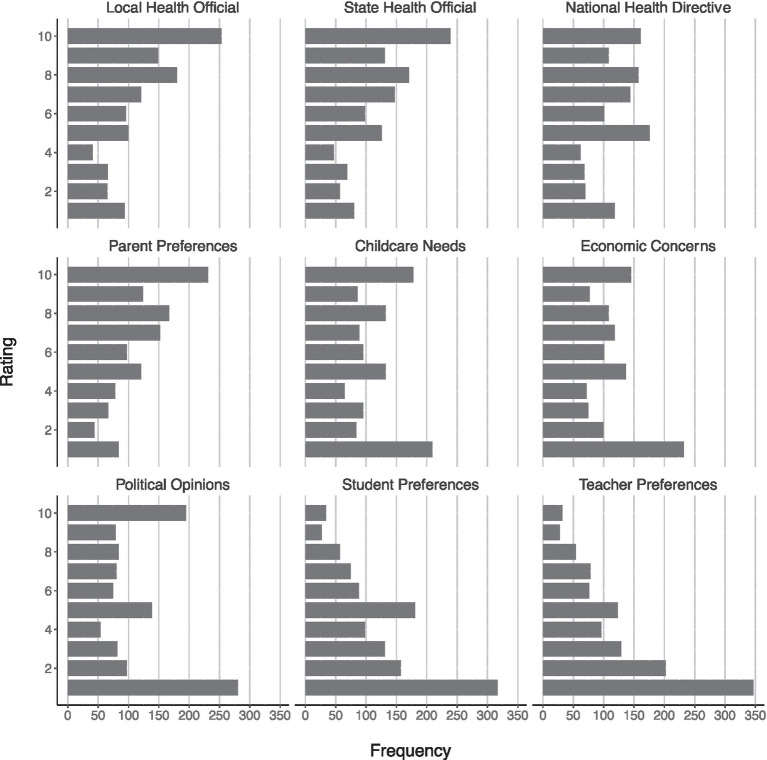
Participants’ ratings of selected influences on mode of instruction.

We asked all participants to rate selected factors according to the degree to which they believe they were influential for deciding the mode of instruction their school chose using a 10-point Likert-type scale. The ratings indicated that the participants felt local (*Mdn* = 7, *IQR* = 4), state (*Mdn* = 7, *IQR* = 4), and national health authorities (*Mdn* = 6, *IQR* = 4) as well as parents’ preferences (*Mdn* = 7, *IQR* = 4) were the most influential factors. These were followed in perceived influence by childcare needs (*Mdn* = 5, *IQR* = 6), economic concerns (*Mdn* = 5, *IQR* = 6), and political opinion (*Mdn* = 5, *IQR* = 6). Student (*Mdn* = 3, *IQR* = 4) and teacher preferences (*Mdn* = 3, *IQR* = 4) were perceived as least influential (see Figure 1). All participants also rated the degree to which they felt acclimated to their current mode of teaching on a scale ranging from 1 *not at all acclimated* to 10 *completely acclimated*, which resulted in a median rating of 6 (*IQR* = 3). We examined whether participants’ perceptions of being acclimated varied as a function of whether they had professional development over the summer to prepare them for the fall or not using a Mann–Whitney U test and no significant differences were found.

Given the variety of teaching modes we anticipated the participants being in, we asked them to report whether they felt their health was at risk due to their teaching environment. Similar to the previous ratings scales, participants responded using a 10-point scale. The median response was just above the mid-point on the scale (*Mdn =* 6) suggesting a tendency for teachers to perceive that their health was indeed at risk. Moreover, the interquartile range for the ratings was relatively large at 7, and nearly half of the participants (46.3%) provided a rating of 7 or higher, which suggest many teachers perceived their teaching environment to be a serious risk to their health. Spearman correlations were computed in order to determine whether the participants’ perceptions of risks to their health were related to their reports of well-being (Table 3). Although the coefficients describing the correlation between the participants’ perception of risk and the well-being measures (Positive Emotion, Health, and Depression) were statistically significant, they were indicative of only extremely weak relationships.

**Table 3:** Correlations among the well-being scales, the Depression scale, teachers’ reports of perceptions their health was at risk, and teachers’ reports of changes in efficacy.

	Health	Depression	Health at risk^a^	Teaching efficacy^a^
Positive Emotion	0.50	0.67	−0.25	0.26
Health		−0.37	−0.21	0.12
Depression			0.24	−0.24

All coefficients significant at *p* < 0.001.

a Spearman coefficients for health at risk pairings, otherwise Pearson coefficients.

### What Were Music Teachers’ Perceptions of the Resources Available to Them and Expectations Made of Them as Compared to Other Teachers at Their Schools?

**Figure 2:**
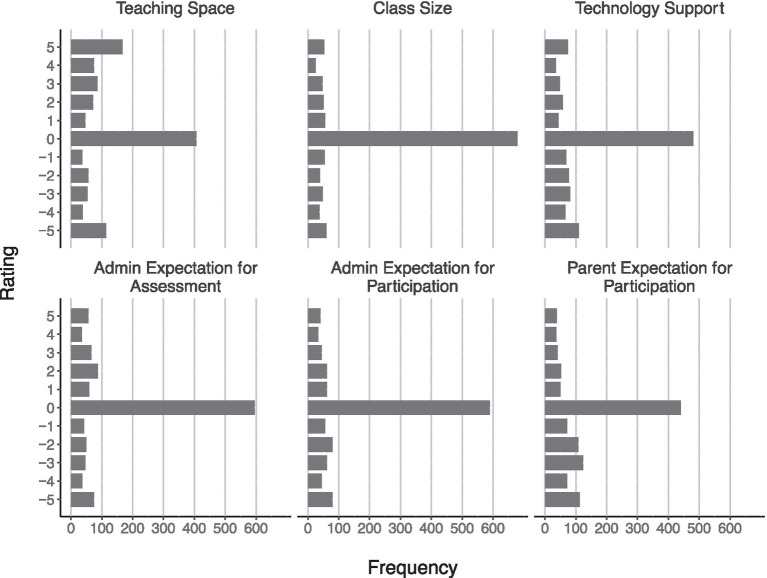
Participants’ ratings of resources and expectations in comparison with other teachers at their schools.

Participants rated the degree to which (1) the resources they had to work with (i.e., teaching space, technology support, and class size) and (2) the expectations made of them regarding student participation and assessment of learning were comparable to that of the other teachers at their schools. For these reports, the participants rated several items used an 11-point rating scale with three anchor points: −5 *my situation is worse than other teachers in my school*, 0 *my situation is similar to other teachers in my school*, and +5 *my situation is better than other teachers in my school*. The vast majority of participants provided a rating of 0 for each of these items, suggesting they did not perceive differences between themselves and the other teachers at their schools (Figure 2). However, there was substantial variability across ratings for these items as well. For example, trends in the data suggest that, overall, participants perceived having slightly better teaching space resources, slightly worse access to technology support, and slightly lower expectations for participation, which perhaps suggests that parents were less concerned with music than other subjects.

We also asked whether participants were reassigned to teaching duties outside of their subject area. Although it was a relatively small proportion of the sample, many participants (*n* = 174, 15.1%) reported teaching outside of their typical subject area. Of these participants, most were either serving some type of general support capacity for their school (*n =* 57, 33.7%; e.g., study hall, academic tutoring, teacher’s aid, working with counselors, lunch, and recess) or re-assigned to teach a class in another academic area (*n =* 52, 30.8%; e.g., art, speech, US history, social studies, and physical education). In addition, nearly 20% of these teachers (*n =* 33) were serving as substitutes in other teachers’ classes and about 13% were re-assigned (*n =* 20) to another type of music class.

### How Have Music Teachers’ Perceptions of Their Teaching Efficacy Changed Since the Spring of 2020 and Do They Perceive Any Positive Outcomes Emerging From the Changes in Their Instructional Practices?

Participants also reported whether they perceived a change in their teaching efficacy since the Spring of 2020 by rating an item paired with an 11-point scale with three anchor points: −5 *my situation is worse than other teachers in my school*, 0 *my situation is similar to other teachers in my school*, and +5 *my situation is better than other teachers in my school*. Overall, their reports suggested a slight improvement in teaching efficacy (*Mdn* = 1, *IQR* = 5). However, there was also a good deal of variability among the ratings. Spearman correlations were used to examine whether the music teachers’ perceptions of changes to their teaching efficacy were related to their reports of well-being (Table 3). Although the coefficients were statistically significant, they were indicative of only extremely weak relationships.

**Figure 3:**
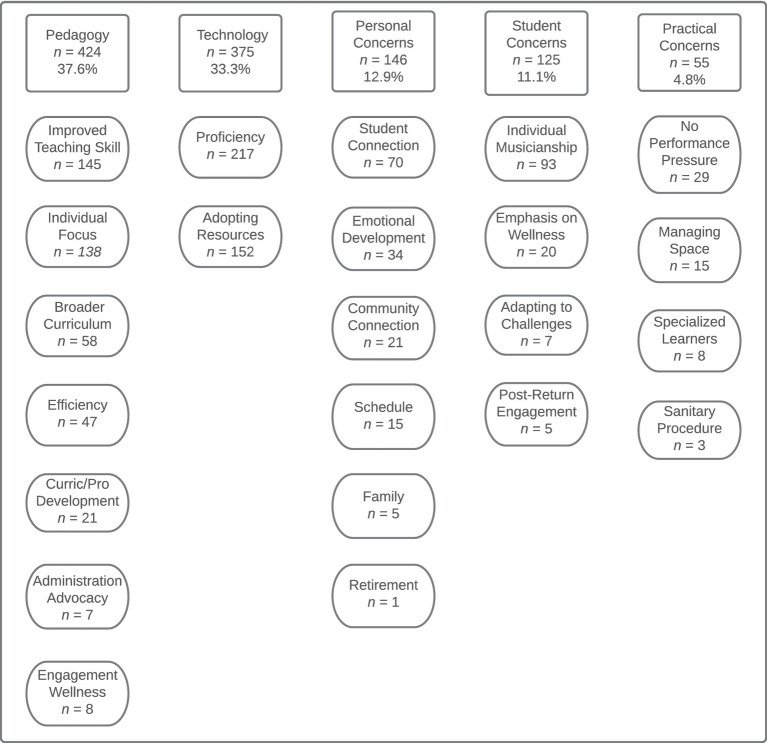
Codes and categories summarizing the positive outcomes the participants reported resulting from pandemic-induced changes in instruction.

**Figure 4:**
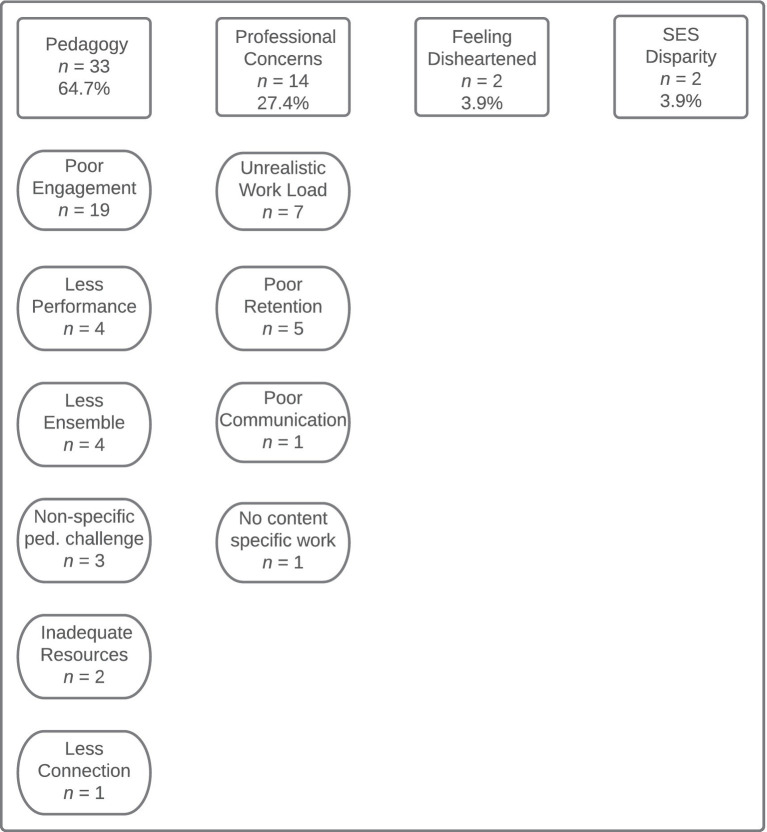
Codes and categories summarizing the negative outcomes the participants reported resulting from pandemic-induced changes in instruction.

We also wished to learn whether any positive outcomes may have resulted from any changes in instructional practices the participants may have instituted since the start of the pandemic. We received 970 responses (73% of participants) to the open-ended item addressing this topic. However, some participants wrote in information regarding negative outcomes in addition to or instead of positive comments. As such, we first categorized the specific contents of these statements according to whether they represented positive (*n =* 1,125) or negative outcomes (*n =* 51). Then, the statements were coded according to the topics contained in each response, resulting in 24 codes for the comments addressing positive outcomes which were subsequently reduced into 5 higher-level categories and 12 codes for the comments addressing negative outcomes that were ultimately reduced into four higher-level categories (see Figures 3, 4).

The participants’ comments regarding positive outcomes were grouped according to the following topical categories: (1) pedagogy (*n* = 424, 37.6%), (2) technology (*n* = 375, 33.3%), (3) personal concerns (*n* = 146, 12.9%), (4) student concerns (*n* = 125, 11.1%), and (5) practical concerns (*n* = 55, 4.8%). Comments regarding positive influences on pedagogy and positive adaptions of technology were the most prevalent, accounting for more than 70% of all positive comments when considered together. The comments subsumed by the pedagogy category also include the most specific codes (seven codes). Of these specific codes, the most prominent comments addressed general issues improvement in teaching skills due to pandemic-related constraints, methods for engaging individual students, opportunities for broadening curricular goals, and a general sense of increased efficiency in teaching. The comments regarding the positive influence of technology were less varied with only two codes representing either participants’ newly gained proficiency with some technology skill/tool or their adoption of a resource they found helpful.

Although less frequently represented, the participants’ comments regarding positive impacts upon personal (six codes) and student (four codes) concerns were quite varied. Most of comments categorized as personal concerns indicated that participants felt the pandemic led them to experience satisfaction through personal connections with students and had led them to reconnect with/develop a deeper emotional connection with their work. Although there were four codes to represent the comments with the student concerns category, most of the comments addressed the participants’ perceptions that some students were benefitting through greater degrees of individual musical development. The least frequently represented category among all of the positive comments was practical concerns. Of these comments, most were coded as referring to either a sense of relief due to reduced performance pressure or reports of having adequate space to rehearse in a socially distanced manner.

Reports of negative outcomes that have resulted from changes in instructional practices since the start of the pandemic were much less common—which is to be expected given that the wording of the item they responded to addressed only positive outcomes. The negative comments were grouped according to four topical categories: (1) pedagogy (*n* = 33, 64.7%), (2) professional concerns (*n* = 14, 27.4%), (3) feeling disheartened (*n* = 2, 3.9%), and (4) socioeconomic disparity evident among students (*n* = 2, 3.9%). The comments about negative outcomes were most frequently about pedagogy or professional concerns. In regard to pedagogy, the most common issue participants reported was a lack of student engagement. Comments reporting negative outcomes that were categorized as professional concerns were most about a sense of unrealistic workload or poor program retention.

**Figure 5:**
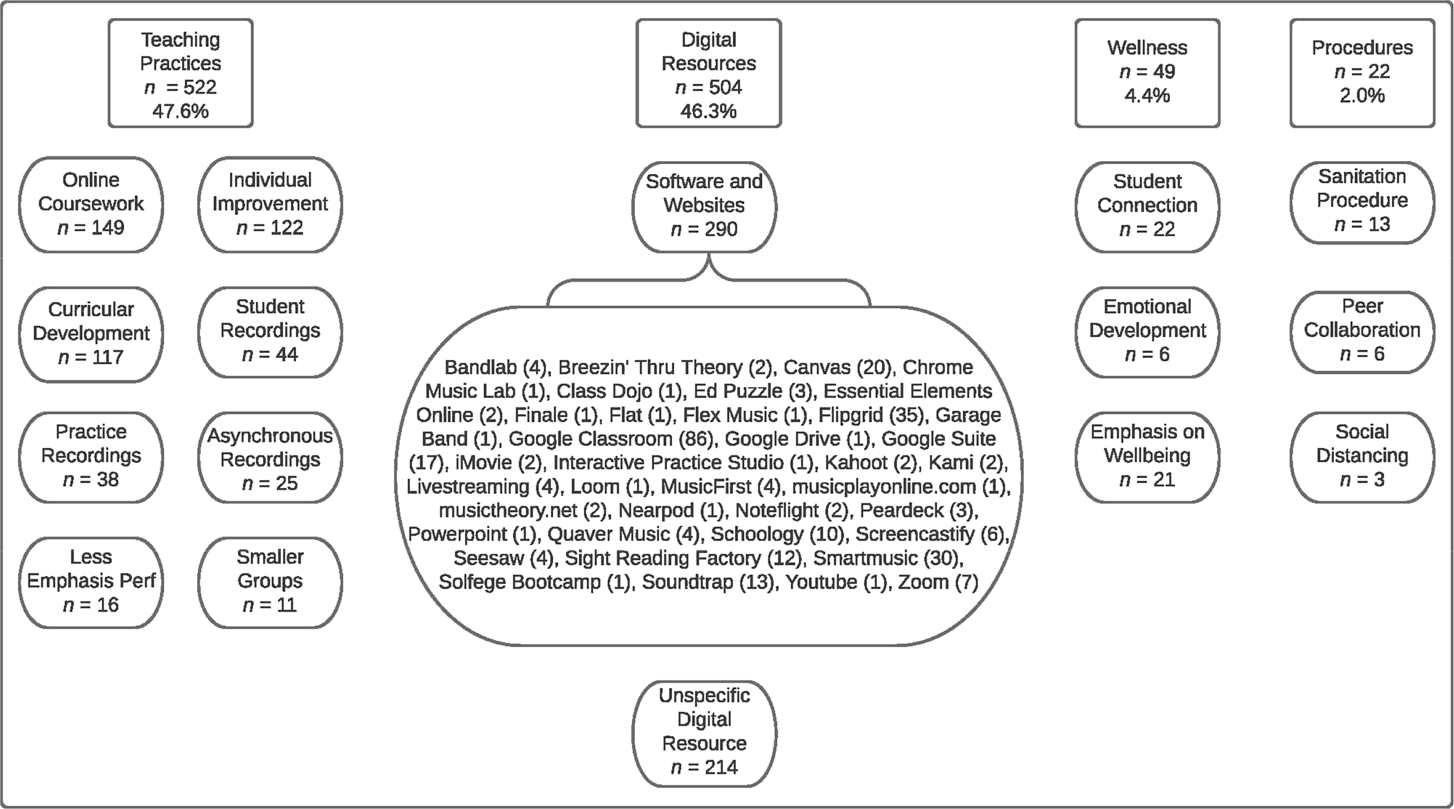
Codes and categories summarizing the pandemic-induced changes in instruction that would like to continue to implement after the pandemic.

Last, we asked the participants whether they would maintain any changes in instructional practices the participants may have instituted once the pandemic-imposed disruptions ceased. We received responses from 947 (71.4% of the total sample) participants who would like to continue to implement some of the changes enacted as a result of the pandemic. As with the previous set of comments, our analyses involved an initial coding of all the statements, followed by focused coding to develop categories. Ultimately, we coded 1,095 comments resulting in 16 codes which were subsequently grouped within four categories: (1) teaching practices (8 codes), (2) digital resources (2 codes), (3) wellness-related issues (3 codes), and (4) procedures (3 codes; see Figure 5).

The most prominently represented categories were teaching practices and digital resources, accounting for nearly 94% of all comments. The teaching practices category subsumed codes referring to a wide range of pedagogical and curricular topics. The most common codes included in this category were indicative of the participants’ desires to continue to include some component of online coursework as well as a focus on individual student improvement and independence once the pandemic conditions cease. Perhaps relatedly, the teachers also commented rather frequently on their interests in continuing to develop and broaden their curricular offerings. Other codes indicated the teachers were interested in continuing to use recording technologies for assessing students, guiding students’ practice, and delivering instruction. Although not frequently cited, some teachers also hoped to continue to emphasize performance expectations less and work with students in smaller groups. In regard to digital resources, many teachers described a generic interest in using technology of some sort and these comments are represented in the “unspecific digital resource” code. However, many participants also described the specific technological tools they would like to continue to use. The full list of the various applications and Web sites the teachers mentioned as well as how often each was mentioned is included in Figure 5. The remaining two categories generated from the responses to this item represented the teachers’ interests in maintaining some degree of emphasis on wellness matters and the new procedural aspects of their work.

## Discussion

### Music Teachers’ Perceptions of Their Well-being

The well-being of the participants in this study was negatively impacted by the pandemic-induced conditions they experienced while teaching during Fall 2020 at levels that are concerning. Their Positive Emotion and Health scores were significantly lower than published norms. Further, their well-being in these areas was not trending in a positive direction as their scores were significantly lower than those reported by a sample from the same population only 5 months earlier, at the end of the previous school year (Miksza et al., 2021; Parkes et al., 2021). In addition, 77% of these music teachers reported at least some level of Depression, with nearly a quarter (24%) of their scores falling into the *severe* or *extremely severe* classifications. Clearly, these music teachers were struggling and this is supported by the findings in emerging research (e.g., Cornett, 2020; Cheng and Lam 2021).

Significant relationships were found between two individual characteristics of the participants—years of teaching experience and area of teaching emphasis—and measures of well-being. Years of teaching experience was positively related to Positive Emotion scores. In other words, music teachers who had taught for a longer period of time tended to have higher Positive Emotion scores. Years of teaching experience was also negatively related to Depression scores; the more the years of experience, the lower the level of depression. It may be that seasoned teachers, who had been through other trying times in their lives and careers, were able to rationalize that although the particular challenges brought on by the COVID-19 virus were unlike any they had experienced previously, their prior experience of surviving other trials may have provided them with a sense of resilience in terms of believing that they would get through this period of time too.

A significant relationship was also noted between the Health scale and area of teaching emphasis. Orchestra teachers reported better health than general music, band, or other music teachers. Choir teachers’ Health scores were significantly lower than other music teachers. While years of experience was positively related to Health, it only accounted for 1% of the variance in scores. While Depression scores also varied by area of teaching emphasis, they only accounted for a very small (0.9%) portion of the variance. Some of these findings may be explained by the differing nature of music teaching in each of these areas. Playing a string instrument does not require one to expel air through the mouth as does playing wind instruments that comprise the majority of band instruments, or singing, which is synonymous with performing in a choir and a frequent general music activity. Therefore, orchestra (string) teachers and their students may not have been as impacted by requirements to wear masks to limit the spread of aerosols as were music teachers and classes in other areas of music education. String students could continue playing their instruments in a manner very similar to their prior practice. In addition, much of string pedagogy, especially with younger students, is visual. String teachers can easily see the posture and body/instrument relationship, left- and right-hand positions, the position and movement of a bow across strings, etc., whereas many aspects of the production of sound when singing and playing a wind instrument are dependent on processes that take place inside the mouth (e.g., articulation), require an open throat (e.g., airflow), and involve deep breathing, which become even harder to remediate when having to wear masks and maintain an appropriate social distance. It makes sense that music teachers whose pedagogy required greater adjustments and who were intent on having their students continue to perform music, perhaps being reluctant to move too much of their curriculum and instruction to learning outcomes related to creating or responding to music, outcomes that may have been more feasible given remote instruction and/or safety constraints such as masks and social distancing, might have experienced additional stress, resulting in a lower Health score.

No significant findings were found related to school characteristics—school urbanicity, Title 1 status, and school size. The nature of a particular school, at least as measured by these three variables, did not seem to result in variation of Positive Emotion, Health, or Depression scores across the participants in this study. The pandemic created challenges for all music teachers, in all types of schools.

### Modes of Instructional Delivery

The start of the 2020–2021 school year caused a number of challenges for the participants. Only a small portion of these music teachers began the school year teaching face-to-face (16.7%), with everyone else having to teach fully or partially online. A number of the teachers (*n* = 150, 12.5%) had to teach face-to-face and online students simultaneously, a particularly challenging task for any teacher, but perhaps more so for music teachers due to the performance-based nature of many music learning outcomes. Moreover, the vast majority of the participants received less than 5 weeks of advanced notice about the mode of instruction they would need to use, which participants reported as being insufficient (Mdn score of 4 out of 10). This is not enough time to fully plan under normal conditions, let alone when having to develop new pedagogical and procedural strategies for a relatively unfamiliar mode of instructional delivery.

While nearly three-quarters of the participants had received some type of professional development to help them with the pandemic-imposed instructional demands, most were lukewarm in their assessment of how helpful it was (Mdn score of 6 out of 10). Their professional development most frequently focused on the use of general technology (52%) or specific technology-based music applications (29.8%). A small number of these teachers also had the opportunity to learn more about topics such as the well-being of themselves and their students (8.5%), COVID-19 safety (4.6%), and social justice issues (3.6%). It appears that most of these professional development experiences were brief—a week or less—although only about a quarter of the participants provided this information.

An ongoing problem with helping teachers understand how to integrate technology into their teaching and students’ learning is that often professional development in this area focuses on the technology itself with little regard to how that technology specifically connects to disciplinary content or pedagogical strategies for using that technology in ways that help support the achievement of valued learning outcomes (Bauer, 2020). While understanding technology itself is certainly important, that in itself is insufficient for the meaningful integration of technology. For example, a general overview of how to operate Zoom effectively is only part of what teachers need to effectively use that tool to facilitate students’ acquisition of knowledge and skills. Specific pedagogical approaches that align with the affordances of the technology and the disciplinary subject matter are also essential to achieving optimum results. In addition, music teachers have unique needs when working with students online because of the nature of musical sound, which requires higher fidelity than speech, and the current state of online technologies that are commonly available, which due to latency issues do not make it possible for multiple people who are distant from each other to simultaneously engage in musical performance. Finally, all types of professional development designed and delivered to a population of teachers within a school system are frequently oriented toward the general classroom teacher and frequently do not address the specialized needs of music teachers (Bauer, 2007). While the specific details of the professional development experiences of these music teachers are unknown, it is not unreasonable to suspect that the application of the content of the sessions to music education was left to the teachers themselves.

Not only was it a challenge to plan for the start of the school year, for many of the participants (60%) their mode of instruction changed after the year began. Most often this resulted in a move to additional online teaching. When this happened, it occurred suddenly, with more than a third of the music teachers indicating they were given only a week to make the transition. Overall participants did not feel they received adequate time to make the switch (Mdn score of 4 out of 10). This situation likely contributed further to the stress and anxiety these teachers experienced.

The teachers reported teaching students (1) face-to-face, (2) online, and (3) face-to-face and online simultaneously, sometimes referred to as HyFlex (Lederman, 2020). It is not surprising that the teachers all felt a single instructional mode—whether face-to-face or online—was better for students than the HyFlex model. These teachers were adapting to new face-to-face safety protocols and procedures, and novel online, technological and pedagogical approaches. That in itself was surely stressful and required substantial effort. But to have to integrate these instructional modes to create a functional class of students physically present and online, at a distance, would be challenging under the best of circumstances. Most teachers felt face-to-face instruction was a better experience, but interestingly 10% of the participants believed the two learning approaches were similar. It would be interesting to know more about the background and experiences of this 10% to better understand why they felt this way while most teachers did not.

Teachers had little input into the decision about the mode of instruction to be used in their school. Local, state, and national health authorities, along with parents, were cited as having the most influence. Their minimal involvement, along with uncertainty surrounding the efficacy of safety protocols, may have contributed to the teachers’ beliefs that their teaching environment posed a risk to their health. A lack of control in any situation can lead to feelings of uncertainty and even fear. When considered in the light of this pandemic and the possibility of getting very sick and even dying, it is not difficult to understand why these teachers reacted as they did.

### Music Teachers’ Perceptions of Resources, Equity, and Teaching Efficacy

Most of the participants indicated that they were treated equitably in terms of how they compared to other educators in their school, although their responses varied greatly. A small number of the participants (15%) reported being asked to help with general school needs (e.g., study hall, recess, and lunch duty) or to teach in another subject area. Importantly, they perceived their teaching efficacy as slightly improved from the previous spring, although, again, there was quite a bit of variability in their responses. Overall, it appears that these teachers were supported in a manner comparable to their colleagues and that perhaps they had adjusted their instructional approaches and/or mindset in a manner that allowed them greater efficacy as educators.

The teachers recognized that there were some positive aspects to their pandemic experiences. They indicated they achieved stronger personal connections with students, which was satisfying, and felt that students were benefitting due to a greater focus on their individual musicianship, likely because group outcomes that are typical of ensembles were not possible. This focus on the individual student—the development of musical independence—was something that they hoped to continue post-pandemic. They also appeared to realize that a broader curriculum was desirable and that technology had value as a learning tool when used appropriately. However, the teachers also expressed concerns about student engagement, heavy workloads, and retention of students in their programs.

Music education in the United States, particularly at the secondary level, has been heavily focused on music ensembles—band, orchestra, and choir. These programs have been very successful by many measures. However, they tend to prioritize group outcomes over the development of individual musicianship. Because traditional ensemble experiences were not possible due to technological limitations when learning moved online, teachers were forced to rethink music teaching and learning at a very fundamental level. Since the pursuit of ensemble outcomes was not feasible, this may have resulted in, at least in some instances, a renewed emphasis on the musical growth and development of individual students. In reality, music teachers should focus on the musical development of each student just as math teachers are concerned about the mathematical development of every student, science teachers work to assure the scientific development of their students, and so on. Of course, such an approach is not to the detriment of ensembles. The stronger each individual is in an ensemble, the stronger the ensemble as a whole will also be. Perhaps, the pandemic will be an event that will result in a rethinking of what is most important about school music education and what types of pedagogical approaches are most appropriate for achieving those outcomes.

For many years, music education leaders have called for a more comprehensive curriculum that included the development of varied musical knowledge and skills (e.g., O’Toole, 2003; Isbell and Stanley, 2018; Webster, 2018), and engagement with diverse genres and styles of music (e.g., Choate, 1968; Powell et al., 2019; Wade and Campbell, 2021). There have also been advocates for the use of technology in music teaching and learning (e.g., Richmond, 2005; Dorfman, 2013; Bauer, 2020). In some cases, the conditions imposed on music teachers and their students provided the impetus for, and in the case of technology an absolute necessity, exploration of these ideas. Again, perhaps, one positive outcome of the pandemic will be a broader music curriculum and adoption of pedagogical approaches that provide students with a more diverse skill set and wide-ranging understanding of music, even once prior forms of music teaching and learning are once again possible. Implementing a broader curriculum is a compelling topic for future research.

### Limitations and Further Research

It is important to recognize that this research is based on a volunteer sample of pre-school to high school music educators in the United States. We used the largest professional organization of music teachers in the United States, in which each state is represented among the 130,000 members (NAfME, 2021), to solicit participation. We do acknowledge that some music teachers in the United States do not belong to the NAfME organization but there was not another alternative to achieve our sample size without the assistance of the organization. We chose a quantitative design and recognize that surveys alone cannot yield the nuance or contextualized detail in the way qualitative research might; however, the strength of our research may be seen in the information garnered from a large sample. For more nuanced contextualization, we recommend interview and qualitative studies be conducted. Our data were gathered at one point in the year, and it is possible that different data would be gathered at other times of the year. Therefore, generalizations beyond this population are not warranted and it is possible that our findings may suffer to some degree from response bias. However, given the similarity between the sample in the current study and that in our previous work (Miksza et al., 2021; Parkes et al., 2021), it appears that representative coverage of the population may have been achieved. Conceptually, this study is also limited due to the lack of a baseline of well-being and depression for this population prior to onset of the pandemic. As a result, it is not possible to determine whether the participants’ worrisome reports of well-being and depression are specifically due to the pandemic. However, given the negative trends identified by comparing the data from this study to our previous work with this population, it seems likely that the pandemic has contributed to prevalence of ill-being among these participants.

Regarding the future research, it would be interesting to investigate whether music teachers are having similar experiences to those in the general teacher population, or if their experiences are markedly more severe. Given that the music teachers did not necessarily report having fewer resources than their colleagues during the pandemic, it may be that other teachers feel similarly. As mentioned, interview or qualitative research may provide more context and certainly the issue of broader curriculum should be examined in depth. Last, it would also be valuable to explore how music teacher identity may interact with the challenges faced during the pandemic. For example, it is possible that a music teacher’s resilience to the obstacles they encountered could vary according to their sense of career commitment and their perceptions of the role they play in their chosen field.

## Conclusion

Teachers are reporting relatively extreme levels of stress as the pandemic-imposed conditions continue to impact their work. The results from the current study suggest that for music teachers in the United States, their well-being has gotten worse from the start of the pandemic in the Spring of 2020 through the start of a new school year in Fall 2020. The reports of depression are particularly concerning. It is clear that the future efforts to identify strategies and resources for providing relief and support for music teachers are critical.

## Data Availability Statement

The raw data supporting the conclusions of this article will be made available by the authors, without undue reservation.

## Ethics Statement

The studies involving human participants were reviewed and approved by the Indiana University Institutional Review Board. Written informed consent for participation was not required for this study in accordance with the national legislation and the institutional requirements.

## Author Contributions

All authors listed have made a substantial, direct and intellectual contribution to the work, and approved it for publication.

### Conflict of Interest

The authors declare that the research was conducted in the absence of any commercial or financial relationships that could be construed as a potential conflict of interest.

## Footnotes

^1^The terms “educator” and “teacher” are synonymous in the United States and used interchangeably throughout this paper.
