# Supplementary material for: The Well-being and Instructional Experiences of K-12 Music Educators: Starting a New School Year During a Pandemic
Source: Front Psychol. 2021 Jul 21;12:701189. doi: 10.3389/fpsyg.2021.701189 (PMC8337047; doi:10.3389/fpsyg.2021.701189)
Supplement: Supplementary file 1 [file Data_Sheet_1.pdf]

Appendix

Questionnaire Items

(Items from the PERMA profiler and the DASS-21 not included.)

Which best describes the grade level(s) of the students you teach?

- Pre-School
- Elementary School
- Middle School/Jr High School
- High School

Which do you consider the primary emphasis area of your teaching?

- General Music
- Band
- Orchestra
- Choir
- Jazz
- Music Technology
- Music Theory
- Piano
- Music History/Appreciation
- Modern Band
- Percussion
- Other (please specify)

How many years have you taught? (including the 2019-2020 school year)

Which best describes your route to teacher certification?

- Traditional college or 4-5 year university teacher preparation program
- An alternate or non-traditional teacher preparation program
- I am not currently certified to teach

Which best describes your highest degree in music education?

- Bachelor's degree
- Master's degree
- Ed.D.
- DMA/DME
- Ph.D.

Which best describes your ethnicity? (select all that apply)

- White or Caucasian
- Black or African American
- Hispanic or Latino
- Asian or Asian American
- American Indian or Alaska Native
- Native Hawaiian or other Pacific Islander
- Middle Eastern
- Other (please specify)

What is your self-identified gender?

Which best describes the setting of your school?

- Urban
- Suburban
- Rural

Is your school a Title I school?

- Yes
- No

Which best describes the size of your school?

- Small (fewer than 500 students)
- Medium (501 to 1200 students)
- Large (1201 students or more)

If you know, roughly what percentage of the students in your school receive free or reduced lunch?

Which best describes the majority of the student population at your school?

- White or Caucasian
- Black or African American
- Hispanic or Latino
- Asian or Asian American
- American Indian or Alaska Native
- Native Hawaiian or other Pacific Islander
- Middle Eastern
- Other (please specify)

What form of instruction did you use to begin this school year?

- Fully face-to-face
- Fully online
- Hybrid—teaching the same students, part of the time face-to-face and part of the time online
- Mixed—teaching some students face-to-face and other (different) students online
- Simultaneous—teaching face-to-face and online students at the same time
- Other

How long before the beginning of the academic year did your school decide on how instruction would be delivered at the beginning of the school year?

- 1 week
- 2 weeks
- 3 weeks
- 4 weeks
- 5 weeks
- 6 weeks
- More than 6 weeks

Please indicate the degree to which the following statement is true for you.

	Strongly Disagree Strongly Agree<br>1 2 3 4 5 6 7 8 9 10
I had adequate time to prepare to teach mu classes once my school made the decision about how instruction would be delivered this fall.	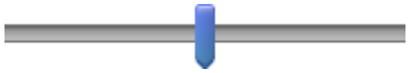

Has your form of instruction changed since the beginning of this school year?

- Yes
- No

What form of instruction are you currently using?

- Fully face-to-face
- Fully online
- Hybrid—teaching the same students, part of the time face-to-face and part of the time online
- Mixed—teaching some students face-to-face and other (different) students online
- Simultaneous—teaching face-to-face and online students at the same time
- Other

How long did your school take to decide how or whether the form of instruction would be changed?

- o 1 week
- o 2 weeks
- o 3 weeks
- o 4 weeks
- o 5 weeks
- o 6 weeks
- o More than 6 weeks

Please indicate the degree to which the following statement is true for you.

	Strongly Disagree						Strongly Agree			
	1	2	3	4	5	6	7	8	9	10
I had adequate time to prepare to teach my classes once my school made the decision about how the form of instruction would be changed.										

To what degree do you believe the following factors influenced decisions about the form of instruction in your school?

	Strongly Disagree						Strongly Agree			
	1	2	3	4	5	6	7	8	9	10
Teachers' preferences										
Parents' preferences										
Students' preferences'										
National directives from organizations such as the Center for Disease Control (CDC)										
State-level health officials										
Local-level health officials										
Child care needs										
Economic concerns										
Political opinions										

Please indicate the degree to which the following statement is true for you.

	Strongly Disagree									Strongly Agree
	1	2	3	4	5	6	7	8	9	10
I feel that my health is at risk because of my teaching environment this Fall.										

If you are teaching both online and face-to-face in some capacity, which students do you believe are receiving better educational experiences?

- o Face-to-face students are receiving better educational experiences
- o Online students are receiving better educational experiences
- o Face-to-face and online are receiving comparable educational experiences
- o Not applicable. I only teach using one modality (either face-to-face or online)

How do the following aspects of your current teaching situation compare to other teachers in your school

	My situation is worse than other teachers in my school					My situation is similar to other teachers in my school					My situation is better than other teachers in my school
	-5	-4	-3	-2	-1	0	1	2	3	4	5
Teaching space (location and adequacy)											
Class size (number of students)											
Parental expectation for student participation											
Administrative expectations for student participation											
Administrative expectations for assessment of student learning											
Support for the use of technology											

Are you being asked to teach outside of your subject area? If yes, please explain in the box provided.

- o Yes
- o No

Please answer the following questions by selecting from the response options provided.

	I feel much less effective					I feel my effectiveness is the same					I feel much more effective
	-5	-4	-3	-2	-1	0	1	2	3	4	5
Compared to to last Spring when the pandemic began, how effective do you currently feel as a teacher?											

Please answer the following question by selecting from the response options provided.

	Not at all Acclimated					Completely Acclimated				
	1	2	3	4	5	6	7	8	9	10
To what degree have you become acclimated to your current teaching environment?										

Did you engage in any professional development over the summer to help prepare you for your fall teaching assignment?

- Yes
- No

Please answer the following question by selecting from the response options provided.

	Not Helpful at All					Extremely Helpful				
	1	2	3	4	5	6	7	8	9	10
How helpful was the professional development you received for your teaching this fall?										

Please provide a brief description of the professional development experience you received. e.g., What topic(s)? What was the length? Other details you believe will help us understand your experience.

Are there any positive outcomes that have resulted from the changes in your instructional practices due the pandemic?

Are there any changes in your instructional practices that you will maintain once the pandemic is over?
